# CTCF and Its Multi-Partner Network for Chromatin Regulation

**DOI:** 10.3390/cells12101357

**Published:** 2023-05-10

**Authors:** Aylin Del Moral-Morales, Marisol Salgado-Albarrán, Yesennia Sánchez-Pérez, Nina Kerstin Wenke, Jan Baumbach, Ernesto Soto-Reyes

**Affiliations:** 1 Departamento de Ciencias Naturales, Universidad Autónoma Metropolitana-Cuajimalpa (UAM-C), Mexico City 05348, Mexico; 2 Institute for Computational Systems Biology, University of Hamburg, D-22607 Hamburg, Germany; 3 Subdirección de Investigación, Instituto Nacional de Cancerología, Mexico City 14080, Mexico; 4 Computational BioMedicine Lab., University of Southern Denmark, DK-5230 Odense, Denmark

**Keywords:** CTCF, epigenetics, chromatin regulation, histone, demethylases, lncRNAs, TET, KDM, BORIS, CTCF-s

## Abstract

Architectural proteins are essential epigenetic regulators that play a critical role in organizing chromatin and controlling gene expression. CTCF (CCCTC-binding factor) is a key architectural protein responsible for maintaining the intricate 3D structure of chromatin. Because of its multivalent properties and plasticity to bind various sequences, CTCF is similar to a Swiss knife for genome organization. Despite the importance of this protein, its mechanisms of action are not fully elucidated. It has been hypothesized that its versatility is achieved through interaction with multiple partners, forming a complex network that regulates chromatin folding within the nucleus. In this review, we delve into CTCF’s interactions with other molecules involved in epigenetic processes, particularly histone and DNA demethylases, as well as several long non-coding RNAs (lncRNAs) that are able to recruit CTCF. Our review highlights the importance of CTCF partners to shed light on chromatin regulation and pave the way for future exploration of the mechanisms that enable the finely-tuned role of CTCF as a master regulator of chromatin.

## 1. Introduction

Chromatin, a macromolecular complex of DNA, RNA, and proteins, provides a framework for the packaging of genetic material within the cell nucleus. Its organization plays a crucial role in gene expression and is regulated by a diverse array of protein complexes in response to a dynamic code of histone posttranslational modifications and DNA modifications [1]. CTCF (CCCTC-binding factor) is a crucial architectural protein believed to play a critical role in maintaining chromatin organization through its interactions with various protein complexes [2]. Among other functions, CTCF is a versatile protein known to participate in various processes related to the chromatin structure, including insulation [3], alternative splicing [4,5,6], transcriptional activation [7], and chromatin loop formation [8]. It is not clear how CTCF has such a dynamic range of functions; however, the response to this question may lie in the context-dependent interactions of CTCF with several protein partners.

Epigenetic complexes, which regulate histone post-translational modifications and DNA methylation, usually contain enzymes that chemically modify the amino-terminal ends of histones, forming a code that determines the chromatin state through a system of writing, reading, and erasing complexes [9,10,11]. The mechanisms by which epigenetic components are recruited to specific regions of the genome have not been fully understood, mainly due to the lack of DNA binding domains in most proteins with epigenetic functions [12]. This is why CTCF is a fundamental protein since it could be the bridge between many epigenetic factors and the DNA [13]. The importance of CTCF protein–protein interactions is highlighted by BORIS (Brother of the Regulator of Imprinted Sites), the paralogous protein of CTCF. The similarity of DNA-binding domains between BORIS and CTCF suggests they share similar targets in the genome [14]; however, due to the low degree of conservation between their terminal domains, it is believed that they interact with different cofactors, which cause them to have opposite consequences in gene expression and chromatin structure [15,16,17].

In addition, long non-coding RNAs (lncRNAs) have been described as crucial factors in chromatin architecture [18]. Recent evidence indicates that CTCF interacts with several lncRNAs that modulate its recruitment and binding to the DNA. Depletion of CTCF RNA binding domains impairs chromatin loop formation and alters transcriptional profiles [19,20]. Moreover, lncRNAs serve as a scaffold for the interaction of CTCF with other proteins in the form of RNA bridges [21] or could even cause it to detach from its DNA binding sites [22]. Without a doubt, CTCF depends on its interactions with other proteins and nucleic acids to exert a wide range of functions. In this review, we aim to shed light on the role of CTCF partners in shaping the 3D organization and gene regulation of chromatin, specifically those with epigenetic function.

## 2. CTCF Is a Multifaceted Protein

**Figure 1:**
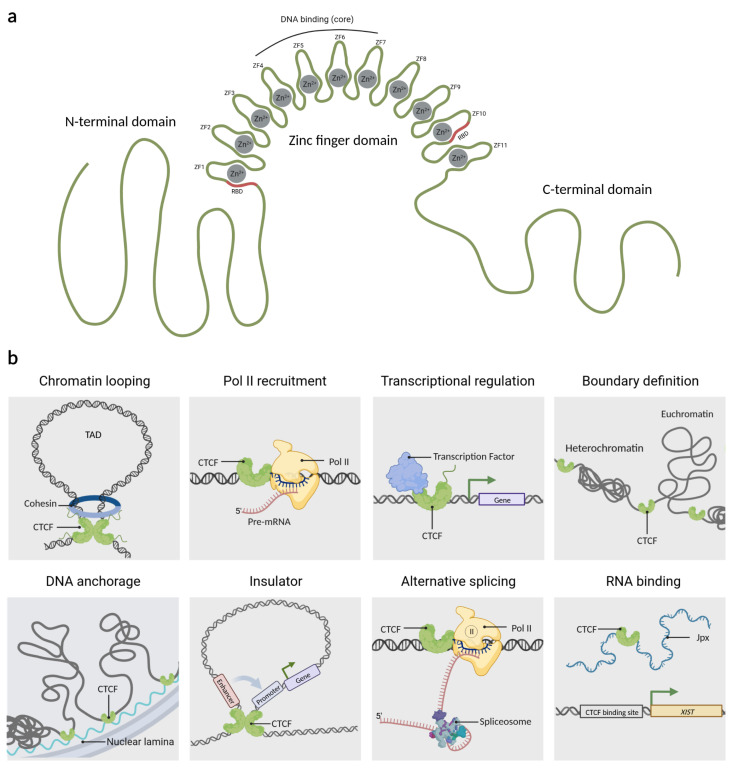
The architectonic factor CTCF. (**a**) CTCF is an 82-kDa protein that contains three domains: an N-terminal region, a C-terminal region, and a central domain of 11 zinc fingers. Moreover, CTCF uses the zinc finger domain cooperatively to bind to DNA. RBD:RNA binding domain, ZF: zinc finger. (**b**) Overview of the wide arrange of CTCF mechanisms of action as: Chromatin looping, RNA Polymerase II (Pol II) recruitment, transcriptional regulation, boundary definition, DNA anchorage, insulator, alternative splicing, and RNA binding, among others. TAD: topologically associated domain. Created with BioRender.com (accessed on 23 April 2023).

Originally, CTCF was described in chickens as a protein that binds to a region upstream of the c-myc promoter. Because that binding site has three regularly spaced repetitions of the sequence CCCTC, the protein was named CCCTC-binding factor or CTCF [23]. Later, it was found that CTCF is a ubiquitously expressed and highly conserved protein in vertebrates [14,24]. CTCF consists of 727 amino acids (aa) distributed in three domains; a zinc finger DNA-binding domain flanked by the intrinsically disordered N- and C-terminal regions (Figure 1a). The DNA binding domain of CTCF has 11 zinc fingers (ZF) which allow it to interact dynamically with the DNA [25,26,27]. CTCF uses different combinations of its ZF to recognize and bind to a variety of DNA sequences, which is why it is considered a multivalent protein [28,29]. However, around 80% of its target sequences contain the core motif 5’-CCACCAGGTGG-3’ that is recognized by ZFs 4 to 7. Unconserved flanking sequences can be recognized by ZF 1–2 or ZF 8–11, which helps to stabilize the CTCF-DNA complex [30,31,32]. A peculiarity of CTCF is that ZF1 and ZF10 have an RNA binding domain (RBD) which is used to interact with several lncRNAs, providing extra anchorage points for the protein [19,31].

CTCF has tens of thousands of genomic binding sites, some of which are conserved between species and tissues [33]. CTCF actions are dependent on its binding site location; which are mainly located in intergenic regions, although they could also be present in regulatory regions such as enhancers, gene promoters, and within gene bodies [34,35,36]. The main functions of CTCF include maintaining topologically associated domains (TADs), acting as a barrier to the spread of heterochromatic structures, and defining the boundaries between euchromatin and heterochromatin, for this reason, CTCF has been coined as an architectural protein [37,38,39,40]. CTCF also regulates DNA anchorage to cellular structures such as the nuclear lamina [37,38], acts as a protein insulator by controlling the interactions between enhancers and promoters [41], and can function as a scaffold protein for transcription factors [42,43,44] and epigenetic factors [45]. Based on the location of the CTCF in other genomic sites, it has also been demonstrated to be involved in processes such as alternative splicing by pausing RNA Polymerase II (RNAP II) binding to alternative exons, thus providing the required temporal context for co-transcriptional spliceosome formation at weak upstream splice sites [4]. CTCF also interacts with lncRNAs which is important for the transcriptional regulation of genes such as Xist, a lncRNA responsible for X chromosome inactivation. For this reason, CTCF has been considered a very versatile protein similar to a swiss army knife. A summary of its functions is shown in Figure 1b.

## 3. BORIS and CTCF-s Highlight the Importance of CTCF Protein–Protein Interactions

**Figure 2:**
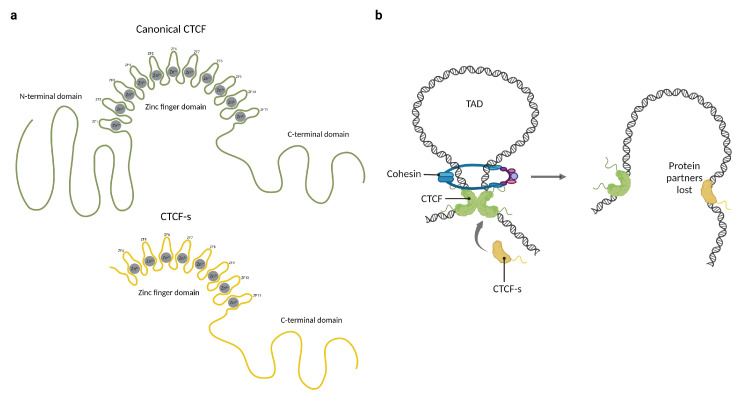
The participation of CTCF and CTCF-short (CTCF-s) in the formation of chromatin loops. (**a**) Representation of the domain distribution of CTCF and CTCF-short (CTCF-s). (**b**) CTCF physically binds to itself to form homodimers which promote chromatin loop formation through cohesin ring protein. CTCF-s competes with CTCF to alter the chromatin architecture and loop formation, mainly because CTCF-s is unable to interact with the cohesin ring. Created with BioRender.com (accessed on 23 April 2023).

The mechanisms underlying CTCF functions are not yet fully understood, but it is probable that most of them depend on interactions with other proteins. One of the better-characterized CTCF protein–protein interactions is cohesin retention. The cohesin ring is a multi-protein complex involved in the formation of chromatin loops [46]. The mechanism of loop extrusion by cohesin involves the translocation of the complex along chromatin fibers, progressively extruding chromatin loops until it encounters a barrier that prevents further movement; such a barrier is frequently a CTCF dimer. In humans, CTCF interacts with SA1-SCC1 subunits of cohesins through its N-terminal domain, fixating the ring in place and establishing topologically associated domains [8,47,48]. Based on this mechanism of action, it has been proposed that upon binding to the DNA, the unbound ZFs and the terminal regions of CTCF might serve as a platform for interaction with other proteins. This hypothesis is supported by the discovery of a shorter isoform known as CTCF-short (CTCF-s), which lacks the N-terminal domain and the first three zinc fingers (Figure 2a). Because they share the core DNA binding domain, CTCF-s competes for the canonical CTCF binding sites and interferes with CTCF–cohesin interactions, causing a disruption in the long-range connection between enhancers and promoters (Figure 2b). Overexpression of CTCF-s leads to increased cell apoptosis in HeLa-S3 cells, but the physiological role of this isoform and its impact on CTCF interactions with other proteins remain uncertain [49].

Similarly, *CTCF* has a paralogous gene called *CTCF-Like* (*CTCFL*), which encodes the protein Brother of the Regulator of Imprinted Sites (BORIS). It is believed that *CTCFL* originated from a duplication event at some point before the evolution of mammals [14]. Unlike CTCF, BORIS is a protein that under physiological circumstances is only expressed in the testis, where it is required for spermatogenesis [50]. Nonetheless, BORIS has gained notoriety recently as a promising drug target because it is aberrantly expressed in several neoplasms and has been related to poor outcomes in cancer patients [51,52].

**Figure 3:**
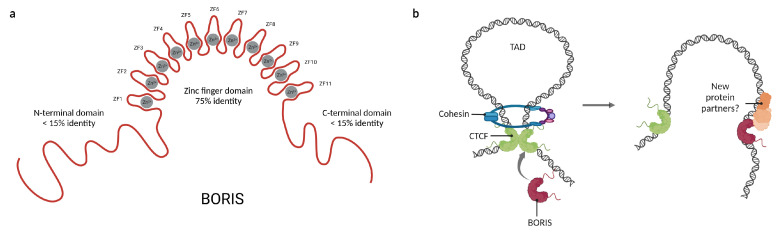
Features and functions of Brother of the Regulator of imprinted sites (BORIS). (**a**) Representation of the domain distribution of BORIS and their percentage of identity with CTCF. (**b**) BORIS can alter chromatin loops by a competitive mechanism with CTCF and its inability to interact with the cohesin ring. Moreover, the recruitment of new protein partners by BORIS could explain the opposite behaviors of CTCF and BORIS. Created with BioRender.com (accessed on 23 April 2023).

CTCF and BORIS share 75% of identity, mainly in their DNA binding domains, suggesting that they might compete for similar binding sites in the genome. Indeed, BORIS has been described to bind to a large subset of CTCF binding sites; however, there are a few differences in the target regions of both proteins [53]. While CTCF binds preferentially to intronic and intergenic regions, BORIS binds mainly to promoters [54,55]. Because the N- and C-terminal domains of BORIS are not conserved (Figure 3a), it has been suggested that BORIS may share binding sites with CTCF, but after binding will recruit different protein partners, interfering with the main functions of CTCF. In this regard, it has been reported that BORIS expression affects transcriptional regulation and the establishment of chromatin loops since BORIS alone is insufficient to recruit the cohesin complex, which is indispensable for CTCF-mediated chromatin loop formation [53,56].

Besides the impairment of chromatin loops, the differences between CTCF and BORIS terminal domains may affect which proteins are recruited upon binding (Figure 3b). Through a yeast two-hybrid assay, it was demonstrated that BORIS binds to a set of completely different protein partners than CTCF [57]. This explains the opposite consequences of their expression in cancer; while BORIS promotes cell proliferation and has been classified as an oncogene [58,59], CTCF is a known tumor suppressor [60]. Moreover, it has been observed that BORIS promotes the expression of some genes that are repressed by CTCF, such as hTERT [61], NY-ESO [17], and H19 [62]. So far, CDH8 and UBF are the only proteins known to bind both CTCF and BORIS [63,64]. A summary of the currently known BORIS protein partners is displayed in Table 1. The former reinforces the importance of CTCF protein–protein interactions for the maintenance of the 3D-chromatin structure and suggests that the terminal domains of these proteins serve as scaffolds for cofactor recruitment. Together, this suggests that the cellular functions of CTCF and BORIS could be defined by their interaction with other proteins.

## 4. CTCF Regulates the Chromatin Structure through Interactions with Several Epigenetic Factors

**Figure 4:**
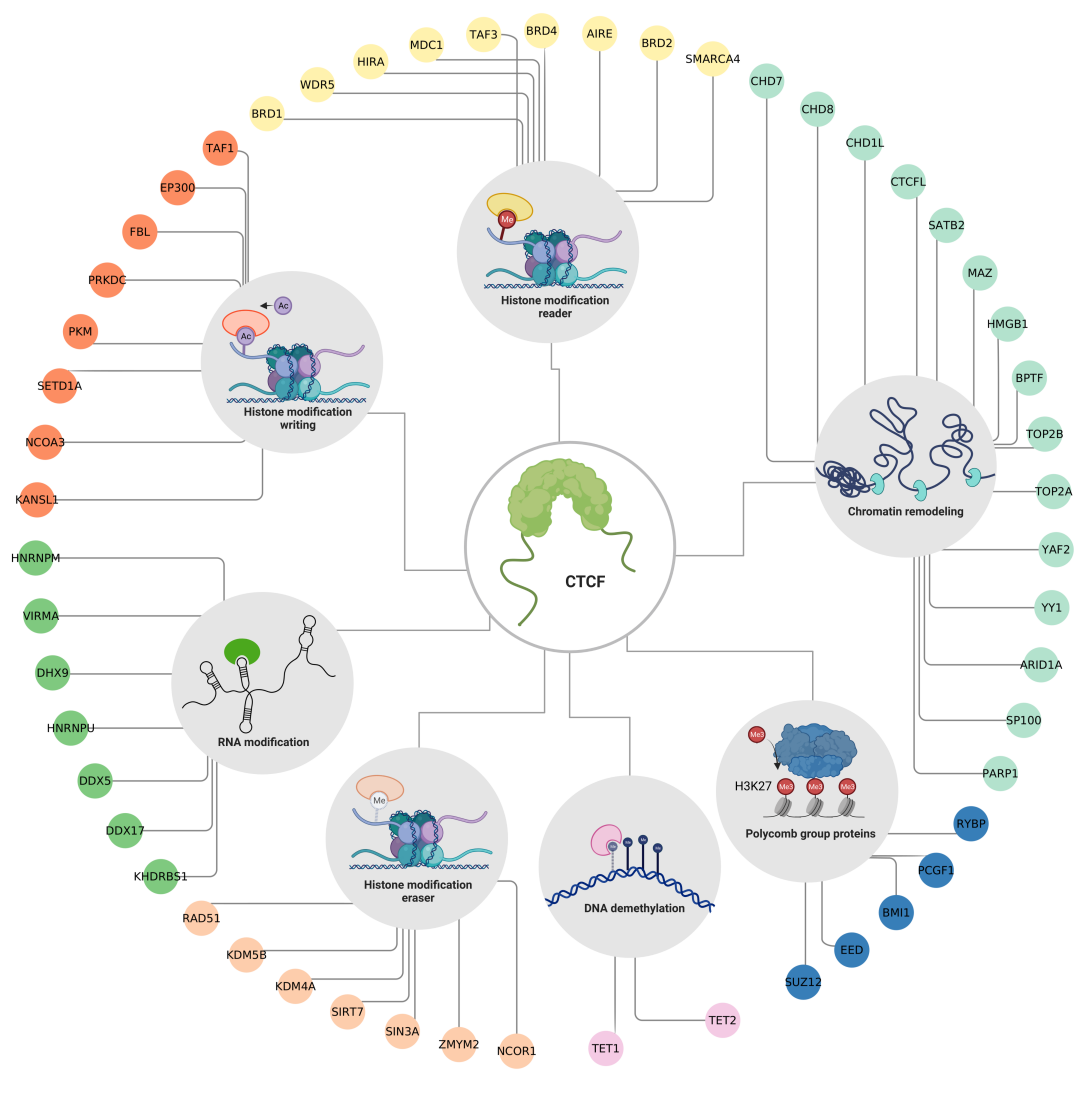
Epigenetic factors that interact with CTCF. The protein–protein interactions between CTCF and other proteins with epigenetic functions. Colors are according to the EpiFactor category that each protein belongs to, as follows: histone modification reader in yellow, chromatin remodeling in mint, polycomb group proteins in blue navy, DNA demethylation in pink, histone modification eraser in salmon, RNA modification in green, and histone modification writing in orange. Created with BioRender.com (accessed on 23 April 2023).

The chromatin status is dynamic and can be regulated by covalent modification of the amino-terminal ends of histones that protrude from the nucleosome and are accessible to enzymes that chemically modify them through a system of writing, reading, and erasing complexes [9]. These modifications correspond to a kind of code that works in conjunction with the DNA sequence to determine the state of the chromatin and establishes and stabilizes gene expression patterns [10]. Because of CTCF’s role as the master regulator of chromatin, it is highly probable that both its actions and DNA recruitment are dependent on the chromatin context. To better understand the interactions between CTCF and other proteins with epigenetic functions, we analyzed data from the literature, as well as the STRING database [69] and the Integrated Interactions Database [70] to find CTCF protein partners (Supplementary Table S1). While many of these partners are transcription factors that use CTCF as a scaffold to shape the chromatin structure [71], CTCF also interacts with other proteins that have epigenetic functions, such as DNA and histone demethylases [21,72,73]. The identification of CTCF protein partners involved in epigenetic processes may provide valuable insights into the complex regulatory mechanisms of chromatin organization and gene expression. To identify these proteins, we filtered our list of CTCF protein partners using the annotations available in the EpiFactors database [13]. The resulting CTCF epigenetic factor targets are shown in Figure 4.

Among these interactions, many of the proteins participate in the shaping of the 3D conformation of the genome such as the DNA helicases CHD7 [74], CHD8 [63] and CHD1L [75], the topoisomerases TOP2A [76] and TOP2B [77], and the components of chromatin remodeling complexes such as ARID1A [78], YY1 [79], YAF2 [42] and BPTF [71]. The former suggests that CTCF works in combination with other remodeling cofactors to establish chromatin domains.

It is also worth noticing that CTCF interacts with several members of the Polycomb group (PcG). These proteins are part of a system that regulates post-translational modifiers of histones, and their action is generally associated with the transcriptional repression of tissue-specific genes. This group has two members, the Polycomb Repressive Complexes 1 and 2 (PRC1 and PRC2). PRC2 is the complex that acts as a writer, as it is responsible for mono-, di-, and trimethylated lysine 27 of histone 3 (H3K27me3). This mark is associated with silenced gene promoters and facultative heterochromatin. H3K27me3 is recognized by PRC1 (reader) that binds to chromatin, monoubiquitinates lysine 119 of histone H2A (H2AK119ub), and prevents transcription by blocking the recruitment of RNA polymerase II [80,81]. CTCF interacts with EED and SUZ12 which are members of the PRC2 complex; a couple of studies have proposed that CTCF could guide the PRC2 complex to gene promoters that are susceptible to repression through H3K27 methylation [82,83]. Furthermore, BMI1, PCGF1, and RYBP are members of the PRC1 complex. Although the biological significance of their interaction with CTCF remains unexplored, a study shows that these proteins may regulate the organization of CTCF-mediated chromatin interactions [84].

Besides PcG proteins and chromatin remodeling factors, CTCF’s relationship with proteins related to histone post-translational modifications are remarkable as well. CTCF interacts with proteins involved in the three stages of histone posttranslational modifications (writing, reading, and erasing). However, we would like to discuss further two particular cases that have not been broadly explored yet; histone and DNA demethylases.

## 5. CTCF as a Modulator of Histone Methylation

Histone methylation is a post-translational modification related to multiple biological functions. Methylation happens mainly in arginine (R) and lysine (K) residues. Arginines can be mono- or dimethylated, and this chemical modification generally potentiates the interaction with other enzymes that modify histone tails [85]. Moreover, lysine residues can be mono-, di-, or trimethylated; these histone marks are associated with either transcriptional activation or repression, depending on the lysine residue. As an example, di- and trimethylation at H3K4 is related to enhanced gene expression, whereas trimethylation at H3K9 and H3K27 is associated with transcriptional repression [10]. Because histone methylation is a covalent modification, it was initially assumed to be stable and irreversible. However, in 2004, the first histone lysine demethylase was characterized, and since then more than 20 enzymes have been described that can remove this covalent modification [86,87].

Currently, histone lysine demethylases (KDMs) are classified into two families based on their chemical mechanism of action: the amine oxidase-like and the oxygenase enzymes [88]. The amino oxidase-like family has two members: KDM1A; the first histone lysine demethylase described by Shi and colleagues in 2004; and KDM1B. These proteins have a common amine oxidase-like domain and are FAD-dependent [89]. KDM1 enzymes can remove mono- and dimethyl groups but cannot demethylate trimethylated lysines, due to their FAD-dependent catalytic mechanism [90]. The oxygenase family is the largest one, with more than 20 JmjC (Jumonji) domain-containing enzymes. These proteins enclose a Fe${}^{2+}$ ion in their catalytic domain and use α-ketoglutarate as a co-substrate [91]. This family is also divided into seven subfamilies (KDM2-8) according to the similarity of their catalytic domain and their substrate specificity [88].

In vitro studies have demonstrated that the simple binding of these enzymes to their substrates is sufficient for the demethylation reaction, suggesting that their recruitment must be tightly controlled in order to prevent aberrant demethylation [92,93,94]. It is not yet clear how the demethylases are directed to specific sites in the chromatin, especially since they lack DNA binding domains. One possible explanation could be that certain transcriptional factors and other chromatin-binding proteins might be responsible for the recruitment of these epigenetic components. KDMs activity could be regulated by protein–protein interactions allowing a dynamical interaction with the chromatin by taking advantage of the “reader” domains present in their binding partners [95,96]. Moreover, it has been suggested that the chromatin environment provides certain selectivity to demethylases since it controls the accessibility of these proteins to their target sites [97]. In addition, it is known that several transcription factors recruit histone demethylases upon binding to their target genes to promote a change in the chromatin state [98,99,100]. However, KDMs’ relationship with CTCF remains partially unexplored.

**Figure 5:**
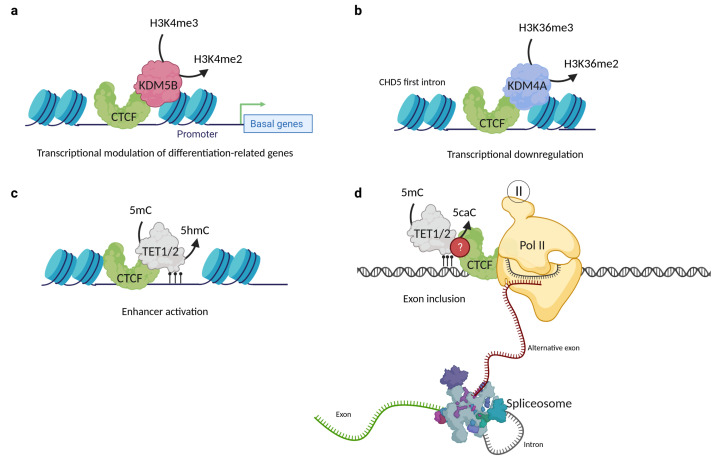
CTCF interactions with histone and DNA demethylases. (**a**) CTCF interacts with KDM5B and regulates the transcription rate of basal/stem cell genes in luminal breast cancer lines. (**b**) The interaction of CTCF with KDM4A is involved in the down-regulation of CHD5 gene expression in MCF7 cells. (**c**) CTCF interaction with TET1 and TET2 proteins is involved in enhancer activation. (**d**) CTCF can also interact with 5caC, which leads to RNA pol II pausing and alternative exon inclusion of the CD45+ gene. CTCF and TET protein–protein interaction is possible but remains uncharacterized for this mechanism. Created with BioRender.com (accessed on 23 April 2023).

Until now, few studies have demonstrated the association between CTCF and histone demethylases; in fact, only two KDM partners have been found. The first was reported in 2014 by Yamamoto et al., who found via co-immunoprecipitation that CTCF formed a complex with the H3K4me3 and H3K4me2 specific demethylase KDM5B. Moreover, when conducting ChIP-seq assays, they discovered that KDM5B sites overlap with those of CTCF in most mammary cancer cell lines, and this overlapping phenomenon correlates with a lower H3K4me3 signal compared to those non-overlapping sites (Figure 5a). The role of the KDM5B-CTCF complex is not clear, but the authors suggest that CTCF takes part in a finely tuned regulation of basal/stem cell genes, such as *ACTG2*, *APOE*, *CTGF*, *FN1*, and *TGFβ2*, among others. The perturbation of these transcriptional changes could promote breast cancer progression [73,101].

Another CTCF histone demethylase partner is KDM4A. The first clue that CTCF could be a KDM4A partner was reported in 2011 by Kang’s group, who performed transfection and immunofluorescence assays and observed that the demethylation frequency of KDM4A was enhanced by the presence of CTCF [102]. This study opened the window to another report in 2018, where co-immunoprecipitation was used to demonstrate that CTCF and KDM4A form a protein complex. Furthermore, it was shown by ChIP-qPCR and ChIP-Re/ChIP-qPCR that CTCF and KDM4A coexist in the first intron of *CHD5*, the promoter of *WRAP53*, and the region located at −1922 bp of the *ASCL2* transcription starting site. The coexistence of CTCF and KDM4A correlates with the reduction of H3K36me3/2 histone modifications at the first intron of *CHD5* and is associated with its transcriptional down-regulation (Figure 5b). Moreover, CTCF or KDM4A depletion mediated by siRNAs leads to the *CHD5* reactivation expression, proposing that both proteins are involved in the negative regulation of this gene. The knockout of KDM4A by CRISPR/Cas9 restored the expression of *CHD5* and H3K36me3 and H3K36me2 histone marks, without disturbing the CTCF localization [72]. Nevertheless, it is currently unknown whether this complex is related to a genome-wide repression or activation and if CTCF might also be one of the key proteins driving the specificity of KDM4A.

To the best of our knowledge, there are no studies evaluating the association between CTCF and other histone demethylases. Nevertheless, ChIP-seq studies demonstrate some overlap between KDM5A, KDM5C, KDM1A, and CTCF, suggesting that CTCF could be involved in their regulation; however, further studies are required to determine the participation of CTCF in the modulation of these enzymes.

## 6. CTCF and the TET Enzymes

DNA methylation is an epigenetic process involving a methyl group transfer to the C5 position of the cytosine to form 5-methylcytosine (5mC). DNA methylation has several functions; although it is generally associated with transcriptional repression; it is also involved in other vital processes, such as genomic imprinting, X chromosome inactivation, and retrotransposon element suppression [103,104]. Similarly to histones, DNA can be demethylated; this process can be accomplished either passively, by simply not methylating the new DNA strand after replication, or actively, by a replication-independent process that involves the ten-eleven translocation (TET) enzymes [105].

The first evidence of the enzyme-mediated DNA demethylation was observed in 2007, with the identification of the Trypanosoma cruzi enzymes JBP1 and JBP2 that are responsible for gene silencing through the hydroxylation and glycosylation of a thymine methyl group (known as J Base). This discovery pointed toward the existence of “eraser” proteins that are in charge of removing DNA methylation [106]. Shortly after, in 2009, when looking for mammalian homologs of the trypanosome thymidine hydroxylases, the three human ten eleven translocation (TET) proteins, TET1, TET2, and TET3 were identified [107]. Nevertheless, the TET proteins were not at a central stage until they were found to oxidize 5mC to 5-hydroxymethyl-cytosine (5hmC) as part of the DNA demethylation mechanism [108,109]. Subsequent reports revealed that TET proteins further oxidize 5hmC to 5-formyl-cytosine (5fC) and 5-carboxyl-cytosine (5caC), both of which are removed through the Base Excision Repair (BER) pathway, thereby completing the demethylation process [108,110].

Because DNA methylation is an epigenetic marker that is essential for correct cellular function and organism development [111,112], TET proteins must be subjected to finely controlled regulatory mechanisms. These enzymes have fundamental roles in epigenetic reprogramming, embryogenesis, development, and tumorigenesis, and it is well-known that their inactivation contributes to the local DNA hypermethylation observed in cancer [113,114]. Apart from catalytic activity regulation, TET1 and TET3 are more likely recruited to their genomic target sites through the direct binding of their respective CXXC domains to the DNA [115]. In vitro binding assays and in vivo chromatin immunoprecipitation assays confirm that these domains can bind CpG-rich oligonucleotides with a slight preference for unmethylated versus methylated substrates [116,117,118]. In contrast, TET2 does not have any obvious DNA-binding domains, and it is therefore potentially recruited through the direct binding of DNA-targeting partners [119]. In fact, it has been demonstrated that the TET2 protein binds tissue-specific transcription factors such as the early B cell factor 1 (EBF1) [120] and WT1 [121,122]. The dynamic expression of DNA-binding factors and their interactions with TET2 can likely concede the tissue-specific and temporal modulation of TET activity on a limited set of genomic loci [123]. Furthermore, interaction with several binding partners is likely to alter the genomic location and stability of TET proteins [124].

Since TET enzymes form protein complexes with other epigenetic components to modify gene transcription, the interaction of these proteins with CTCF is of particular interest. It is known that synchronized fluctuations of DNA methylation, demethylation, nucleosome positioning, and CTCF chromatin binding have an important role in establishing cell-type-specific chromatin states during differentiation. Loss of CTCF in regions such as the boundaries of chromatin loops, promoters, and TADs can be associated with the spread of DNA methylation and demethylation, and can be linked to the down-regulation of adjacent genes. A hierarchical interaction between cytosine modifications, nucleosome positioning, and DNA sequences controls CTCF binding and regulates gene expression [125,126].

It has been proposed that CTCF binding to low methylated regions could mediate local DNA demethylation through TET recruitment [127]. The first evidence was an oscillating 5hmC pattern observed around the binding sites of CTCF in mouse embryonic stem cells, which suggests that accessibility and 5hmC deposition could be related to CTCF binding [128]. The genomic co-localization of CTCF, TET1, TET2, and 5hmc was probed by co-immunoprecipitation assays on 3T3-L1 and HEK293T cell lines and correlated with enhancer activation on differentiated cells through the facilitation of the hydroxymethylation of DNA [129]. This concludes that CTCF directly interacts with the TET enzymes and promotes the DNA hydroxymethylation of enhancers driving adipocyte differentiation (Figure 5c). Nevertheless, the relationship between CTCF and TET demethylases is not only relevant to cell differentiation processes, since a study in 2016 revealed that dynamic TET1 and TET2-catalyzed DNA oxidation stimulates CTCF-dependent alternative splicing in human lymphocytes. This study found that CTCF directly interacts with 5caC in vitro and that this mark was strongly associated with alternative exon inclusion [6]. Moreover, a study demonstrated that 5caC could reinforce CTCF binding to the DNA (Figure 5d). These findings suggest that the TET mediated-induction of 5caC is a potential way to regulate CTCF binding and further reinforces the idea that there is a close relationship between CTCF and the TET proteins [130]. More studies are needed to better describe the exact functions that DNA oxidation plays in transcriptional regulatory events; additional explorations will be required to define the way in which CTCF binding is associated with 5caC in vivo.

Taken together, the above information suggests that CTCF could interact, directly or indirectly, with histone and DNA demethylases; it is still unknown whether these complexes are related to repression, activation, or other transcriptional processes.

## 7. Long Non-Coding RNAs as Non-Protein Partners of CTCF

Long non-coding RNAs (lncRNAs) have emerged as important regulators of chromatin structure and gene expression. They act as scaffolds, guides, or decoys that recruit chromatin modifiers to specific genomic regions, mediate higher-order chromatin organization, and influence gene expression [131]. LncRNAs have been demonstrated to play a critical role in the formation and maintenance of chromatin domains, such as TADs. In this context, lncRNAs have been found to interact with chromatin-associated proteins, CTCF for instance, to modulate their function and impact on chromatin structure and gene regulation [19,132]. Recently, lncRNAs have been identified as key regulators of CTCF [20]. CTCF interacts with RNA through the RNA-binding domains in ZF1 and ZF10. Some studies have even reported a consensus sequence for RNAs that bind to CTCF, and it has been suggested that it could have around 5000 potential RNA partners in the genome [21,133].

LncRNAs contribute to the functions of CTCF by recruiting it to specific genomic sites, modulating chromatin loops, and regulating the formation of TADs. One of the most studied cases is CTCF-mediated Xist transcriptional repression. Xist is a lncRNA involved in X chromosome inactivation. CTCF represses Xist expression by binding to its promoter; however, Jpx is a lncRNA that binds to CTCF and removes it from the *Xist* promoter, allowing its expression and subsequent X chromosome inactivation [19,134]. Recently, it was found that Jpx can also compete for CTCF binding sites in the DNA, altering the loop formation and the overall conformation of the chromatin [22]. The interplay between CTCF and other RNA-binding proteins is also important for the maintenance of TADs. As shown in Figure 4, CTCF interacts with several RNA-binding proteins. Among them, DDX5 is an RNA helicase involved in many steps of RNA-related processes, such as alternative splicing, miRNA biogenesis, and RNA unwinding [135]. It has been described that both DDX5 and the lncRNA steroid receptor RNA activator (SRA) interact with the CTCF-cohesin complex and stabilize it. Such an interaction is required for the insulation activity of CTCF [136].

Several other lncRNAs have been identified to interact with CTCF and modulate its function. HOTTIP, for instance, can recruit CTCF to specific genomic regions and promote TAD formation [137]. Similarly, GATA6-AS1 contributes to TAD formation by forming an RNA-DNA triplex and interacting with CTCF [138]. LncRNAs also regulate gene expression through the recruitment or detachment of CTCF [139,140,141]. PACERR recruits CTCF and p300 to promoter regions to activate gene transcription through histone acetylation [142]. LncRNAs have also been associated to increase protein stability; for instance, the lncRNA ELDR inhibits CTCF degradation by the proteasome, increasing protein levels without modifying transcript levels. Table 2 shows known interactions between CTCF and lncRNAs, along with the putative function of the complexes.

Overall, lncRNAs represent an exciting new area of research in the field of chromatin biology and gene regulation. The interaction between lncRNAs and CTCF offers a new level of complexity to the already intricate network of molecular interactions that govern gene expression and chromatin architecture.

## 8. Conclusions and Final Remarks

CTCF is a nuclear factor that is involved in several chromatin-related processes, including transcriptional regulation, three-dimensional chromatin topology, and epigenetics. Part of its relevance lies in its versatility, as shown in this review, CTCF relies on a broad network of protein and RNA partners to achieve its different tasks. The role of the protein partners is clear upon comparison with CTCF–s and BORIS. In the first case, the lack of the N-terminal domain leads to the loss of the most studied CTCF interacting partners, the cohesin complex. The second case is more complex, since BORIS binding to the DNA has completely opposite consequences than CTCF, besides sharing a high degree of identity at their DNA binding domains. Most of this could be explained by their interactions with different protein partners through their unconserved terminal domains. There is current research going on in this regard, and without a doubt, the study of the interplay between CTCF and BORIS in cancer will help to understand CTCF’s role in chromatin organization and other epigenetic processes.

Chromatin is finely organized inside the nucleus through a complex system that has not yet been elucidated. As mentioned previously in this review, many of the epigenetic factors that help to establish and maintain chromatin structure lack DNA binding domains; thus, it has been hypothesized that their action should rely on other proteins. CTCF is capable of binding to thousands of sites in the genome, and due to the flexibility of its DNA binding domain, it is considered a multivalent protein. In this review, we demonstrate that CTCF interacts with a wide array of epigenetic factors which suggests that it could serve as a scaffold for the assembly of different protein complexes. Nevertheless, the logistics involved in partner election, the impact of each complex, and the crosstalk between different partners is an exciting point of view that is worth further study.

Since epigenetic markers such as histone and DNA methylation are highly dependent on the chromatin context, the interaction between CTCF and different components of the epigenetic complexes is interesting. So far only a few protein–protein interactions between CTCF and other epigenetic factors have been fully characterized, and in most cases, the studies have been conducted on a specific gene or promoter; thus, genomic scale experiments could be helpful to identify the overall impact and localization of the complexes.

LncRNAs add another layer of complexity to the CTCF-mediated chromatin regulation. Currently, the role of most of these complexes in biological processes remains unknown. However, several studies hint towards the existence of a broad CTCF-RNA interaction network. The role of some of these complexes has been discussed here; among them, Jpx is the most remarkable example due to its ability to detach CTCF from its binding sites. Further studies will help to understand the role of CTCF-lncRNA interactions.

Because of CTCF’s versatility, it could likely function as a scaffold for many of the epigenetic complexes required for a proper genomic organization. Without a doubt, there are still undiscovered mechanisms for CTCF; the study of this protein could aid to understand the complex mechanisms that regulate chromatin organization and gene expression.

## Figures and Tables

**Table 1:** Known protein–protein interactions of BORIS. Proteins that were experimentally validated to interact with CTCF as well are labeled in red.

Protein Types	Protein	Complex Function	Experimental Evidence	References
Chromatin-associated proteins	PRMT7	Arginine methylation to control imprinting.	Immunoprecipitation.	[65]
	CTCF	Unknown function in spermatogenesis.	In situ proximity ligation assay. Immunoprecipitation	[50,53]
	BAG6 SET1A	Transcriptional activation of c-myc and BRCA1.	Yeast two-hybrid assay	[57]
	POGZ SRCAP	Unknown	Yeast two-hybrid assay	[57]
	TBP	Transcriptional activation of MAGE-A1.	Pull down assay.	[66]
	SP1	Transcriptional activation of NY-ESO-1.	Immunoprecipitation. Pull down assay.	[66,67]
Transcription factors	ELF2	Unknown	Yeast two-hybrid assay	[57]
	HCFC2, HCFC1			
	MGA			
	TLK2			
	NFAT5			
	ZNF518			
	ATF7			
	MKL2			
	Ku70	DNA damage repair.	Immunoprecipitation	[68]
DNA Binding proteins	**UBF**	rDNA transcriptional regulation.	Immunoprecipitation.	[64]
	**CHD8**			
Signaling proteins	CSTA FHL2	Unknown	Yeast two-hybrid assay	[57]

**Table 2:** Long noncoding RNAs (lncRNAs) that are known to directly interact with CTCF.

lncRNA	Function	References
HOTTIP	CTCF recruitment and TAD formation	[137,140]
PACERR	Recruits CTCF and p300 to promoter regions.	[142]
JPX	Jpx binds to CTCF consensus regions causing a shift in chromatin loops. It is also involved in X chromosome inactivation.	[22,139]
DLGAP1-AS2	Reduced binding of CTCF to target genes.	[140]
GATA6-AS1	May contribute to TAD formation. Forms an RNA-DNA triplex.	[138]
ELDR	Inhibits CTCF degradation by the proteasome.	[143]
SH3PXD2A-AS1	Recruits CTCF to inhibit the expression of target genes.	[141]
CDKN2B-AS1	Recruits CTCF and EZH2 to silence target genes.	[144]
LINC00346	Prevents CTCF binding to the *c-Myc* promoter	[145]
H19	Mediates the interaction between CTCF and Vigilin to regulate IGF2 imprinting.	[146]
Firre	Anchorage of the X chromosome to the nucleolus.	[147]
CCAT1-L	Modulates chromatin loops.	[148]
SRA	Estabilizes CTCF-cohesin complex.	[136]

## Supplementary Materials

The following supporting information can be downloaded at: https://www.mdpi.com/article/10.3390/cells12101357/s1, Table S1: Protein-Protein interactions for CTCF.
